# Improved performance of optical phased arrays assisted by transparent graphene nanoheaters and air trenches

**DOI:** 10.1039/c7ra13154b

**Published:** 2018-02-23

**Authors:** Yubing Wang, Lei Liang, Yongyi Chen, Peng Jia, Li Qin, Yun Liu, Yongqiang Ning, Lijun Wang

**Affiliations:** State Key Laboratory of Luminescence and Applications, Changchun Institute of Optics, Fine Mechanics and Physics, Chinese Academy of Sciences Changchun 130033 P. R. China wangyubing@ciomp.ac.cn

## Abstract

In this paper, high-performance optical phased arrays (OPAs) assisted by transparent graphene nanoheaters and air trenches have been designed and simulated. By directly locating graphene nanoheaters on silicon waveguides, heating efficiency is enhanced by 62.96% compared to conventional structures with 1 μm SiO_2_ overlays, and is further enhanced by a factor of 200% by the presence of air trenches. Thanks to the high thermal conductivity of graphene, a record-high operation speed on the order of 200 kHz is realized. Power consumption for π phase shift is 4.65 mW, approximately half of that of the state-of-the-art OPAs. By introducing air trenches, thermal crosstalk is significantly reduced, resulting in an enlarged fill factor. In addition, a novel beam steering scheme in the *θ* direction is proposed. By applying a 30 mW heating power, a temperature gradient along antennas is generated and beam steering of 2.3° is achieved, satisfying applications such as long-range collision avoidance for autonomous driving.

## Abstract 2

Improved performances of optical phased arrays have been achieved assisted by graphene nanoheaters and air trenches.

## Graphical abstract

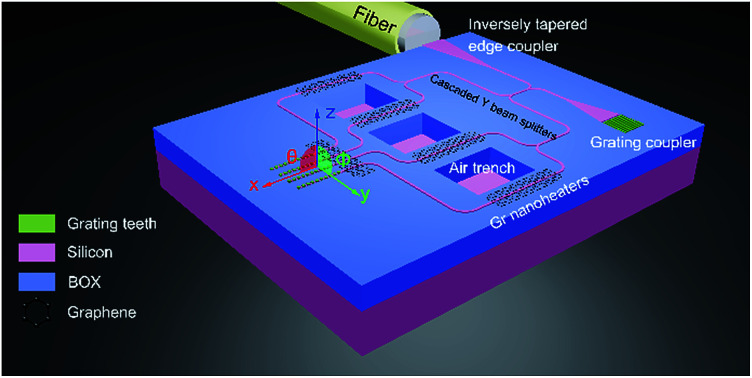

## Introduction

Optical beam shaping and steering is indispensable in many applications such as LIDAR (light detection and ranging) systems for three-dimensional imaging and mapping and free-space optical communication.^1^ Solid-state beam steering is preferable due to its excellent stability, compact size and reduced weight. Recently, T. Cao *et al.* theoretically simulated arrays of gradient Au/Bi_2_Se_3_/Au nanostructures and other metamaterials and achieved continuous beam steering of 18°.^2–6^ However, these exciting results require further experimental research before industrial applications. Optical phased arrays (OPAs) based on arrayed waveguides fabricated *via* CMOS (complementary metal oxide semiconductor) technology have been considered as one of the most promising technologies to realize solid-state LIDAR system due to their acceptable beam steering angle and potentially reduced cost.

**Fig. 1:**
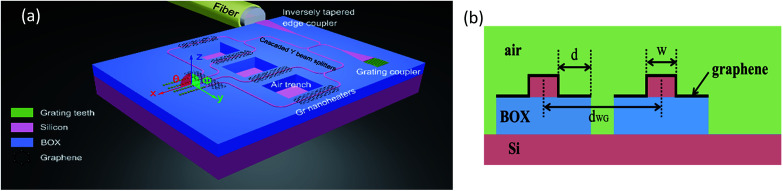
(a) Schematic of the proposed structure (not to scale). (b) Schematic cross-section of the phase-shifting region.

OPAs have been intensively studied in numerous publications.^7–16^ Beam steering in *ϕ* direction (*i.e.* direction perpendicular to waveguide array, as shown in Fig. 1(a)) is achieved by applying a fixed phase shift between adjacent antennas (also referred as emitters) according to the far-field Fraunhofer diffraction, while beam steering in *θ* direction (*i.e.* direction along waveguide array) is accomplished either by wavelength tuning using the dispersive nature of grating or by fabricating two-dimensional (2D) OPAs.

Generally, Si-based OPAs are thermo-optically tuned and the phase shift is determined by1
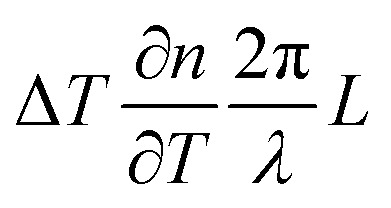
where Δ*T* is temperature difference, 
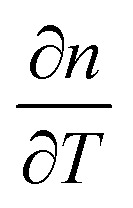
 is thermo-optic coefficient of Si, *L* is heating length and *λ* is wavelength in vacuum.^7^

Metal heaters are widely used in silicon thermo-optic phase shifter.^12,13,16^ However, due to strong optical absorption, metal heaters must be separated from waveguides by thick (∼1 μm) overlay such as SiO_2_, making metal heaters power-inefficient. In addition, poor heat conductivity of SiO_2_ hinders heat transfer, prolonging heating and cooling time. Recent breakthrough in silicon OPAs utilizes an adiabatic bend to insert p–n–junction heaters into Si waveguides.^7,8,10,15^ However this approach not only requires careful design, cutting-edge fabrication technique but also may result in optical loss due to Si doping and the adiabatic bend.

As mentioned above, 2D beam steering requires either wavelength tunable laser diode or 2D OPAs. First, it is challenging to design and fabricate chip-integrated semiconductor laser diode with high power, narrow linewidth and wide tunable range. Wavelength-tunable lasers based on silicon platform have only been realized as proof-of-concept demonstrations.^1^ Moreover, wavelength tuned OPAs are generally fed by edge coupling,^7,9,12,16^ resulting in large coupling loss (>3 dB), due to the relatively small dimensions of Si waveguides compared to optical fibers. Furthermore, although 2D OPAs can achieve 2D beam steering using single wavelength, the major disadvantages are the dramatically increased on-chip power consumption, design and fabrication complexity, and more sophisticated driving circuits.^1,17^

To conclude, it is imperative to realize thermo-optic OPAs with high power-efficiency, fast operation speed and ease of design and fabrication, and a novel low-power beam steering scheme in the *θ* direction without wavelength tunning.

Graphene, a layer of carbon atoms arranged in hexagonal lattice, has received tremendous research interest since its first realization.^18–20^ Experimental results show that graphene has a uniformly high transmission of 97.7% for wide optical spectrum, from 300 nm to 2500 nm.^21^ The transparent nature of graphene allows us directly locating graphene on Si waveguide without any materials in between. Thus heat generated from graphene could transfer directly to Si waveguide, making graphene nanoheaters more power-efficient.^22^ In addition, the thermal conductivity of graphene is measured to be 5300 W m^−1^ K^−1^,^23–25^ over 10^3^ times higher than that of silica and air, which are 1.38 W m^−1^ K^−1^ and 0.023 W m^−1^ K^−1^, respectively. Such high thermal conductivity facilitates heat conduction in the heating and cooling process, making graphene nanoheaters friendlier for high-speed operating situation. Moreover, graphene is compatible with CMOS fabrication process,^26^ making graphene nanoheaters readier for large-scale photonic integration circuits (PICs).

In this paper, we demonstrate improved performance of OPAs assisted by graphene nanoheaters. We show that heating efficiency of the graphene contact heating structure is improved by 62.96% compared to conventional structure with upper cladding. Key parameters including thermal crosstalk, operation speed and power consumption are simulated and the proposed structure is optimized accordingly. In addition, a novel beam steering scheme in the *θ* direction is demonstrated.

## Simulation and design

### Device structure

Schematic of the proposed structure is shown in Fig. 1(a). We only draw four channels for clarity. The cross-section of the phase-shifting region, composed of Si rectangular waveguide, air trenches and graphene transparent nanoheater locating directly on the surface of waveguide, is schematically shown in Fig. 1(b). The substrate is an SOI (silicon-on-insulator) substrate with intrinsic 220 nm top Si and 2000 nm buried oxide (BOX). The choice of this standard SOI substrate mainly depends on commercial availability and compatibility with CMOS PICs foundries, imec-ePIXfab for example. Three independent etching steps are required to manufacture the passive elements: a full etch of 220 nm Si for waveguides, cascaded beam splitters, tapers and air trench windows, a second of 50 nm Si for the grating couplers and a third of 2000 nm silica for the air trenches. Large area graphene sheet is transferred onto Si waveguide by the well-established transfer methods.^27–29^ Graphene nanoheaters are patterned by standard optical lithography followed by oxygen plasma etching. The width of graphene nanoheater is (2*d* + *w* + 440) nm. Throughout this paper, area of the patterned graphene nanoheaters is 100 μm^2^ and heating power applied to graphene nanoheater is 1 mW, unless otherwise mentioned.

### Optical field simulation in Si waveguide

Though only single-layer atom thick, graphene has uniformly strong light absorption for near infrared light (∼2.3%).^21^ When evanescently coupled with Si waveguide, the absorption coefficient of graphene could be further enhanced.^30^ Fortunately, thanks to the relatively low density state of graphene, optical absorption could be eliminated by appropriate doping of graphene. According to the Pauli blocking effect, if the doping level of graphene is high enough that the Fermi level of graphene exceeds 
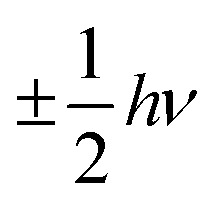
 regime, where *hν* is energy of incident photons, graphene becomes totally transparent.^31–34^ Moreover, numerous publications have discussed methods of uniform doping of graphene, including gate doping,^35^ ion doping^36^ and chemical doping.^37,38^ Therefore, the imaginary part of the refractive index of graphene is assumed zero. In addition, since the thickness of single-layer graphene is 0.34 nm, far smaller than the height of Si waveguide, we could reasonably believe that graphene has negligible influence on optical field distribution. Thus in the optical field simulation, we simplified the model by neglecting the presence of graphene.

The far-field emission pattern of an OPA equals to the far-field pattern of one emitter, or the “envelope”, multiplied by an array factor.^7,10^ In order to obtain large field of view (FOV), the envelope should be as wide as possible. Since the width of the envelope is reversely proportional to the width of emitter, narrow waveguides are preferable.^9^ Thanks to the large refractive index difference between Si and BOX (∼2), the propagation loss of optical field is negligible for waveguide widths greater than 500 nm. Therefore, we choose *w* = 500 nm for simulation, enabling single-mode condition, low propagation loss and wide envelop.

**Fig. 2:**
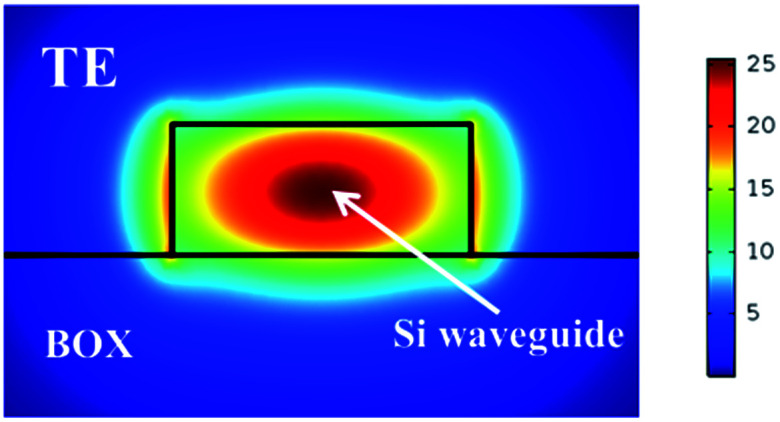
Optical field distribution for TE mode. The width and height of Si waveguide are 500 nm and 220 nm, respectively.

Neglecting optical absorption of Si, BOX and air, the real parts of refractive indexes at 1550 nm are set at 3.48, 1.45 and 1.00, respectively. The simulated optical field distribution for transverse-electric (TE) mode is shown in Fig. 2.

### Thermal field simulation in phase-shifting region

**Fig. 3:**
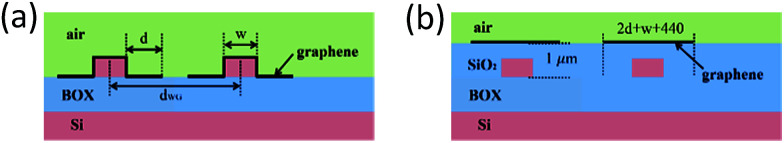
(a) Schematic of graphene contact-heating structure. (b) Schematic of the conventional structure with 1 μm-thick top SiO_2_ cladding layer.

In order to clearly demonstrate the merit of graphene nanoheaters which allows for direct contact heating Si waveguide, we first consider the structure without air trenches, which is schematically shown in Fig. 3(a), as a comparison to the conventional structure with 1 μm-thick SiO_2_ overlay, as shown in Fig. 3(b).

**Fig. 4:**
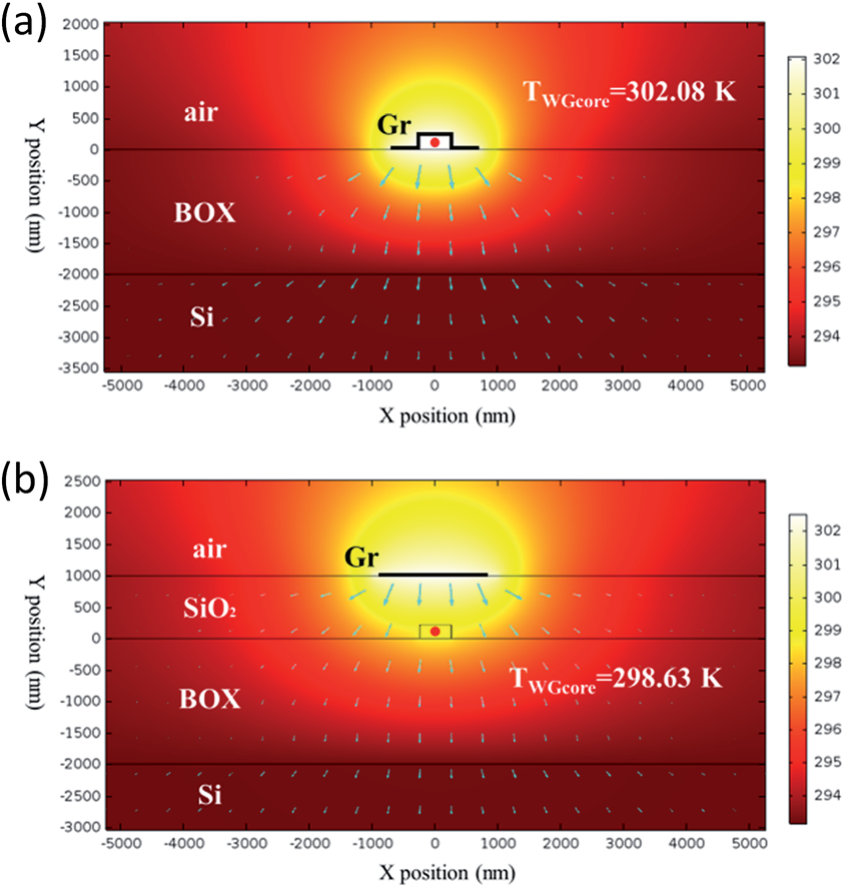
Simulated thermal field distribution of the proposed structure with graphene contact heating (a) and the conventional structure with 1 μm-thick top SiO_2_ cladding layer (b). Blue arrows shows the directions of heat flux.

The thermal field is associated with the heating efficiency of graphene nanoheater. Given a fixed heating power (1 mW), a higher waveguide core temperature (*T*_WGcore_) indicates a higher heating efficiency.^25^ Simulations of thermal field are carried out by a commercial software COMSOL which is based on Finite Element Method. The simulated thermal field distribution of the proposed structure with graphene nanoheaters and the conventional structure are simulated, as shown in Fig. 4(a) and (b), respectively. Here, graphene nanoheater are modeled as thin layers and widths of graphene nanoheaters are 1.94 μm (*d* = 500) for both simulations. Heat sources are defined by boundary heat sources. The thermal conductivity for air, Si, SiO_2_ and graphene are 0.023 W K^−1^ m^−1^, 130 W K^−1^ m^−1^, 1.38 W K^−1^ m^−1^ and 5300 W K^−1^ m^−1^, respectively, and the ambient temperature is 293.15 K.

As shown in Fig. 4, in the conventional structure, *T*_WGcore_ is 298.63 K, corresponding to a temperature elevation of 5.48 K. In the proposed graphene contact-heating structure, *T*_WGcore_ is 302.08 K, corresponding to a temperature elevation of 8.93 K, indicating an enhancement in heating efficiency of 62.96% is obtained. In the graphene contact-heating configuration, heat flux conducts directly to waveguide without any heat dissipation, giving rise to a higher heating efficiency. However, in the conventional structure we can see from Fig. 4(b) that a portion of heat flux, which conducts horizontally in SiO_2_ overlay in the heat deliver process, does not contribute to waveguide heating, resulting in not only lower heating efficiency but also severe thermal crosstalk as discussed below.

**Fig. 5:**
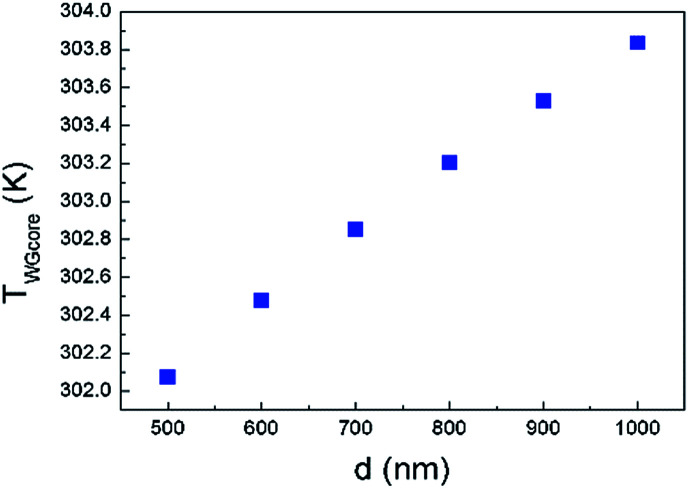
Relationship between *d* and waveguide core temperature *T*_WGcore_.

Relationship between *d* and *T*_WGcore_ is simulated, as shown in Fig. 5. Since the thermal conductivity of Si and SiO_2_ is much higher than that of air, heat flux mainly conducts through BOX and Si substrate. As the width of graphene nanoheater increases, heat flux generated by the additional-width graphene nanoheater conducts horizontally through graphene and contributes to the waveguide heating, leading to higher *T*_WGcore_. It is worth mentioning that although a higher *T*_WGcore_ is obtained with a wider graphene nanoheater, it is less power-efficient (as discussed below) since the heating length is reduced for a fixed area of graphene (100 μm^2^ in our simulation).

**Fig. 6:**
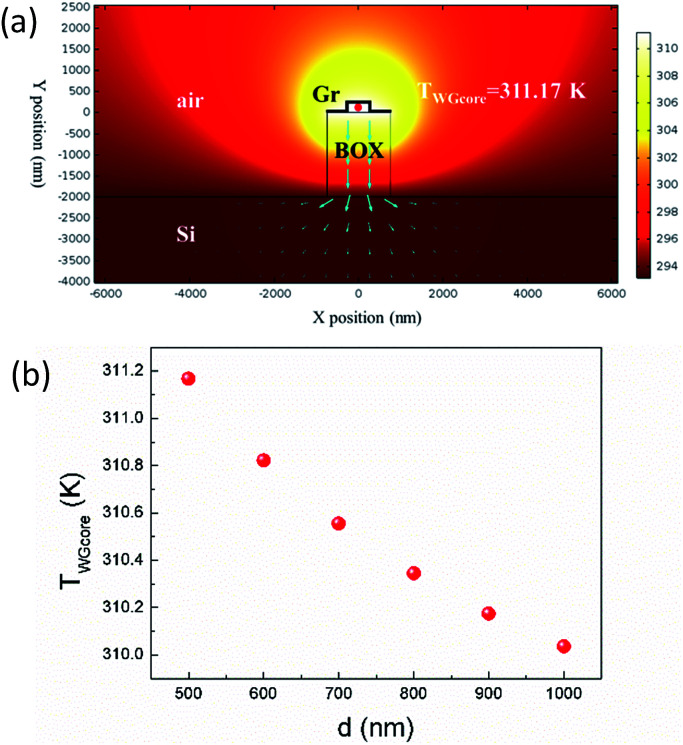
(a) Thermal field distribution with 1.94 μm-wide graphene nanoheater. Blue arrows shows directions of heat flux. (b) Waveguide core temperature as function of *d*.

Recent progress introduces air trenches to thermo-optic phase shifters,^25,39,40^ harnessing the poor thermal conductivity of air, further improving the heating efficiency, trading off response speed to some extent though. The structure is schematically shown in Fig. 1(b), where BOX outside graphene-nanoheater-covering area is fully etched. Simulated thermal field distribution is shown in Fig. 6(a). *T*_WGcore_ is drastically enhanced up to 311.17 K, indicating that the heating efficiency is further improved by a factor of 200% compared to the structure without air trench. *T*_WGcore_ as function of *d* is shown in Fig. 6(b). As can be seen, *T*_WGcore_ decreases with increasing width of graphene nanoheater, showing an obviously opposite trend compared to Fig. 5.

In the structure without air trench, as the width of graphene nanoheater increases, more heat power is generated and then conducts horizontally through graphene and finally contributes to the waveguide heating, resulting in increasing *T*_WGcore_. In the structure with air trench, the above-mentioned process is still valid. However, increasing width of graphene nanoheater enables increasing width of heat dissipation path, leading to a decreasing *T*_WGcore_, countering the effect of additional heating power. As a result, *T*_WGcore_ is affected by the more dominating factor, which is the width of heat dissipation path concluded from our simulation.

**Fig. 7:**
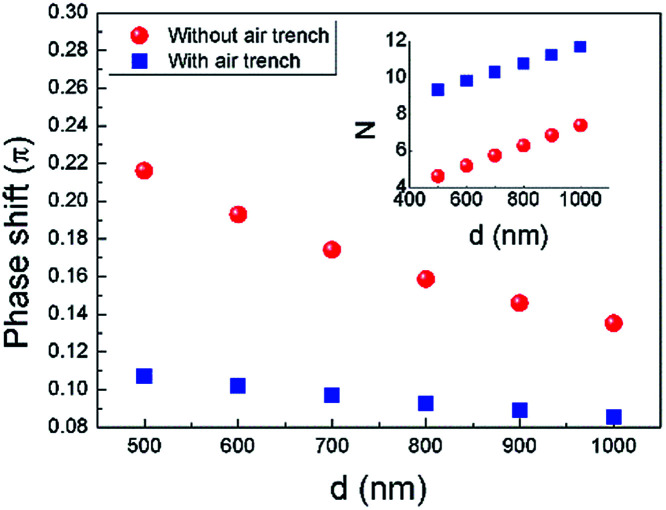
Phase shift of the proposed structure with and without air trench. Inset shows corresponding *N* as function of *d*.

**Fig. 8:**
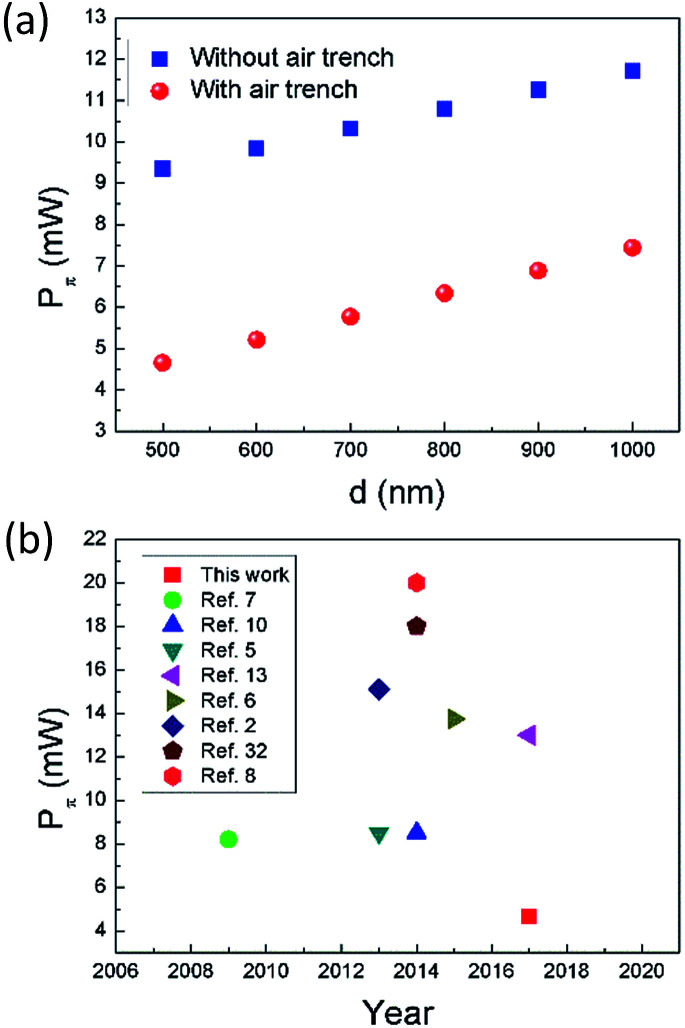
(a) *P*_π_ as function of *d* with and without air trench. (b) Summarization of *P*_π_ reported in references.

Power consumption for π phase shift (*P*_π_) is another important parameter for an OPA's phase shifter, since it is related to the total on-chip power consumption which scales with number of emitters.^1^ Using eqn (1), we calculated the phase shift for the graphene contact-heating structure as function of *d*, as shown in Fig. 7. In order to obtain π phase shift, one may operate multiple graphene nanoheaters in series, where number of nanoheaters *N* equals to reciprocal of phase shift (inset of Fig. 7). However, this method dramatically increases the footprint of the nonradiative phase-shifting region, leading to a lower fill factor. An alternative yet moderately less power-efficient method is operating a single-phase shifter with elevated thermal power. *P*_π_ of the latter case is calculated as shown in Fig. 8(a). A *P*_π_ as low as 4.65 mW is achieved in graphene contact-heating structure with air trench, approximately half of the state-of-the-art OPAs, as shown in Fig. 8(b).

**Fig. 9:**
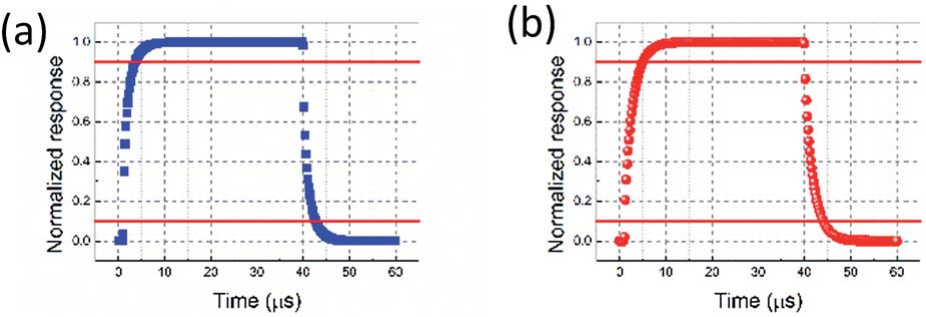
Transient responses in structure without air trench (a) and with air trench (b).

**Fig. 10:**
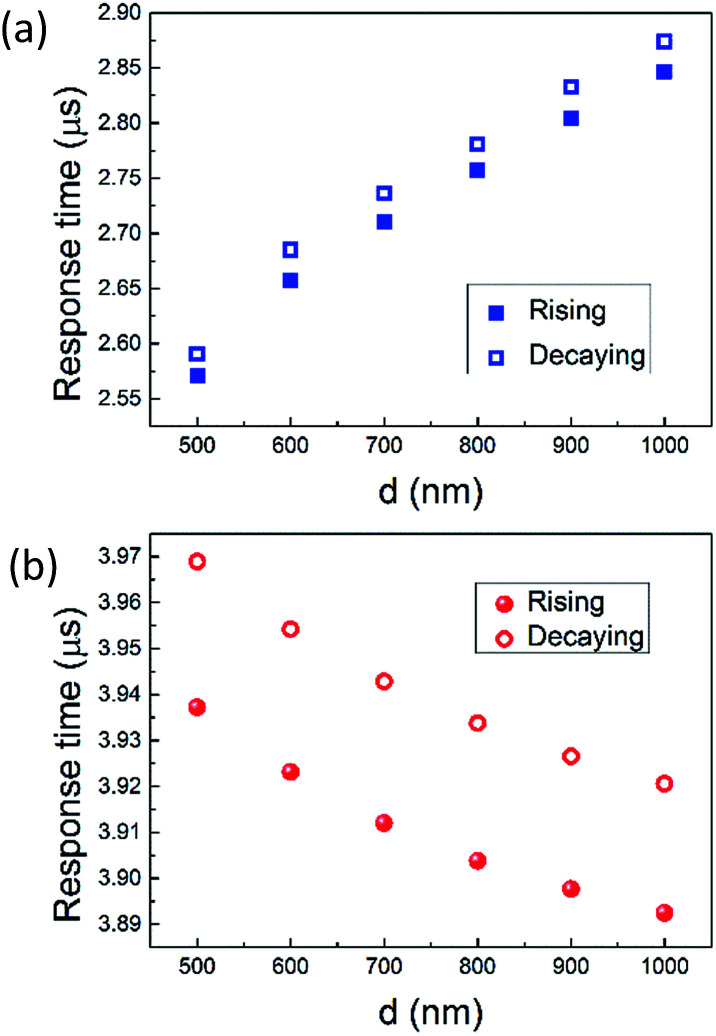
Rising and decaying time as function of *d* in the structure without air trench (a) and with air trench (b).

The transient response is also simulated, as shown in Fig. 9, where *y*-axis is the normalized temperature difference at waveguide core compared to ambient condition (Δ*T*_WGcore_). The 10–90% rising time and decaying time for graphene nanoheaters without air trench are 2.57 μs and 2.59 μs. As qualitatively analysed above, the rising time and decaying time for graphene nanoheaters with air trench are relatively longer, which are 3.93 μs and 3.96 μs, respectively, due to the poor thermal conductivity of air. The response time as function of *d* in both structures are shown in Fig. 10. The response time in the structure without air trench is proportional to *d*, as opposed to that in the structure with air trench. Explanation to this opposite trend is due to the opposite trend of *T*_WGcore_ as function of *d*. Qualitatively speaking, it requires longer time to reach thermal equilibrium for higher *T*_WGcore_. The shortest response time is on order of 5.16 μs, corresponding to an operation speed on order of 190 kHz, which is, to our best knowledge, the fastest thermally tuned OPA. Please note this operation speed is limited by 10–90% response time, unlike ref. 41 in which the operation speed is determined by half-maximum steering angle. Following ref. 41, we would achieve a corresponding operation speed on order of 200 kHz.

**Fig. 11:**
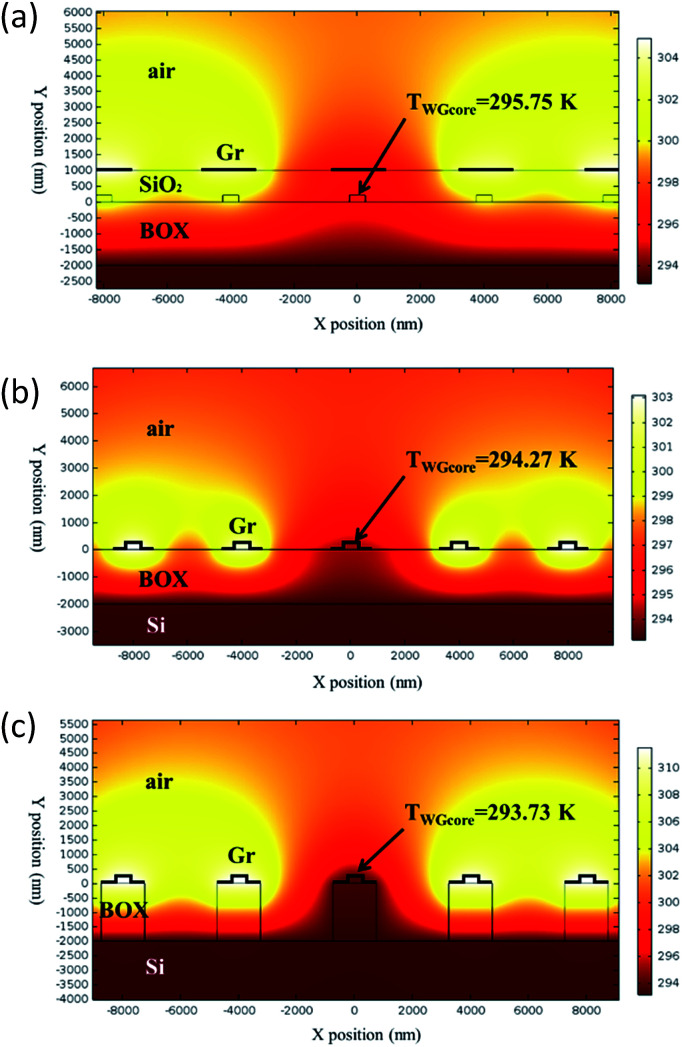
Thermal field distributions of conventional structure with 1 μm-thick SiO_2_ top cladding (a), graphene contact-heating structure without air trench (b) and with air trench (c).

Thermal crosstalk is detrimental for OPAs, especially for the large-scale integrated ones, where each emitter requires an independent thermo-optic phase shifter in order to balance phase difference resulted from fabrication imperfection.^11,16^ Therefore low thermal crosstalk is another design criterion. Thermal crosstalk among emitters is simulated by applying 1 mW heating power to all graphene nanoheaters except for the central one,^16^ as shown in Fig. 11. A higher temperature at the central waveguide means severer thermal crosstalk. The width of graphene nanoheaters are 1.94 μm (*d* = 500 nm) and distances between adjacent waveguides are 4 μm (*d*_WG_ = 4 μm).

**Fig. 12:**
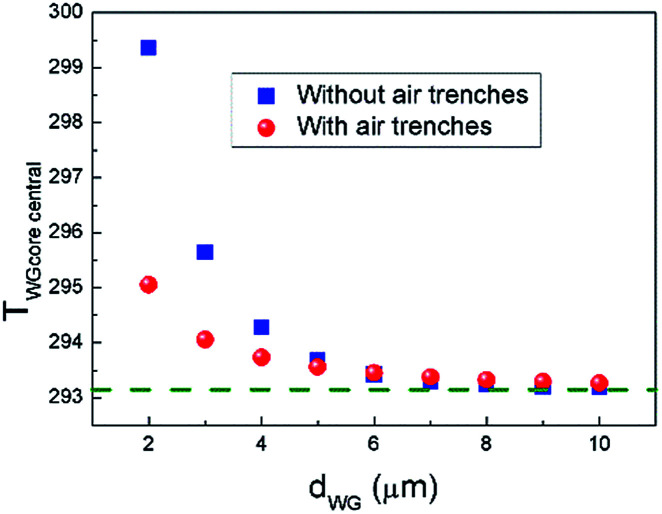
Dependence of temperature at central waveguide core (*T*_WGcore central_) on waveguide pitch (*d*_WG_) with and without air trench. Dashed green line indicates the ambient temperature.

As shown in Fig. 11(a)–(c), the waveguide core temperature in the conventional structure and the proposed graphene contact-heating structures without and with air trenches are 295.75 K, 294.27 K and 293.73 K, respectively, corresponding to temperature elevations of 2.6 K, 1.1 K and 0.58 K at the central waveguides, respectively, indicating ameliorated thermal crosstalk between channels in the graphene contact-heating structures. Thermal crosstalk in the graphene contact-heating structure with air trench is reduced by a factor of 448% compared to the conventional structure. In the conventional structure, heat flux conducts vertically through SiO_2_, heating up the waveguide underneath, as well as horizontally to adjacent waveguides in the heat deliver process, leading to severe thermal crosstalk. However, in the graphene contact-heating structure, heating flux generated by graphene flows directly to the Si waveguide. Furthermore, the presence of air trenches introduces heat isolation among channels that significantly diminishes thermal crosstalk. Dependence of the temperature at central waveguide core (*T*_WGcore central_) on waveguide pitch (*d*_WG_) with and without air trench is shown in Fig. 12. It can be seen that when *d*_WG_ is smaller than 4 μm, the central waveguide suffers from severe thermal crosstalk in both structures, although thermal crosstalk in the structure with air trench is approximately half of that in the structure without air trench. However, when d_WG_ is larger than 10 μm, thermal crosstalk is negligible. It is worth mentioning that the waveguide pitch in the conventional structure is generally on order of 100 μm to reduce thermal crosstalks.^16^ Using graphene nanoheaters, the waveguide pitch can be reduced by one order of magnitude, giving rise to significantly reduced size of OPA chip, footprint of nonradiative phase-shifting area and consequently enlarged fill factor.

### Beam steering in *θ* direction

Achieving wide steering range in the *ϕ* direction requires smaller antenna pitch and is only an issue of nano-processing technology. However, beam steering in the *θ* direction is much more challenging. Generally speaking, in an ideal OPA, beam steering range in *θ* direction should be as wide as possible. However, for some realistic applications, such as collision avoidance for autonomous driving at 100 m distance or longer, beam steering as wide as 2 degree is practically enough since the angle subtended from a pedestrian or a vehicle is merely on order of 1 degree.

In a grating emitter, each grating tooth can be regarded as an antenna. Therefore, by operating a graphene nanoheater parallel to the *y*-axis at the beginning of the antenna array (Fig. 1(a)), a temperature gradient is generated parallel to the *x*-axis, resulting in beam steering in the *θ* direction.

**Fig. 13:**
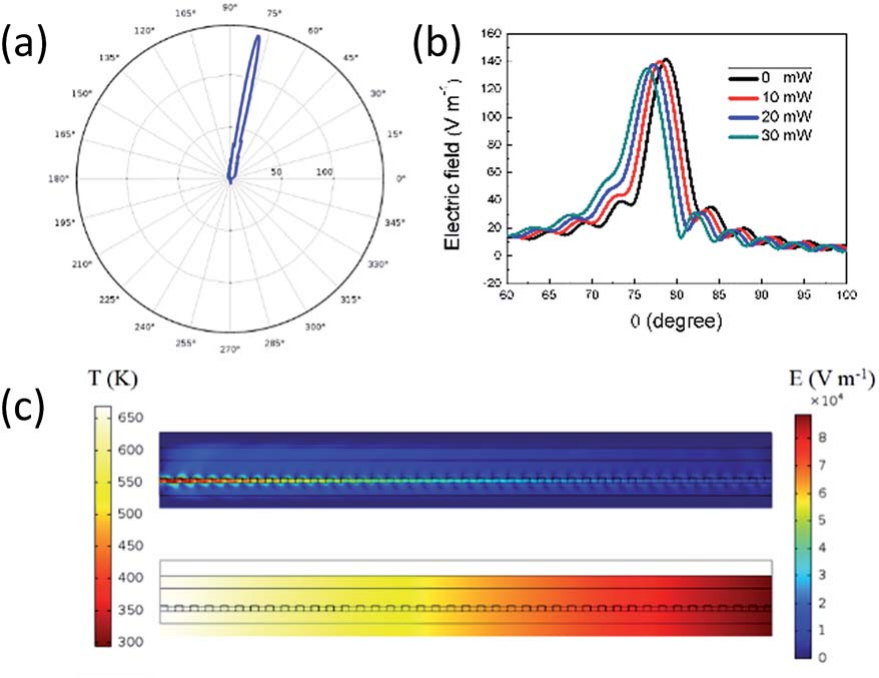
(a) Polar plot of the far-field radiation pattern. (b) Beam steering for 0–30 mW heating power with 10 mW step. (c) Electric field distribution (upper panel) and thermal field distribution (lower panel) corresponding to 30 mW.

The grating pitch and duty cycle modeled in this paper are 600 nm and 50%, respectively. Using 2D symmetry, the polar plot of the upward-radiated far-field pattern is shown in Fig. 13(a), with peak radiation direction at 78.8° and a beam width of 5° (full width at half maximum). Beam steering at various heating power is shown in Fig. 13(b). For 30 mW the peak radiation direction is at 76.5°, indicating a beam steering of 2.3° is achieved. The electric field and thermal field distribution corresponding to 30 mW are shown in Fig. 13(c).

## Conclusion

In this paper, improved performances of OPAs assisted by transparent graphene nanoheaters have been demonstrated. First, assisted by transparent graphene nanoheaters, the heating efficiency is enhanced by 62.96% compared to the conventional structure with 1 μm SiO_2_ overlay, and is further enhanced by a factor of 200% by the presence of air trench. In addition, the operation speed is on order of 200 kHz, twice the previously reported fastest OPA. Moreover, power consumption for π phase shift is as low as 4.65 mW, approximately half of the state-of-the-art OPAs. Furthermore, thanks to the poor thermal conductivity of air, thermal crosstalk is reduced by factor of 448% compared to the conventional structure, leading to footprint of nonradiative area reduced by one order of magnitude and consequently significantly enlarged fill factor. Finally, by applying 30 mW heating power, beam steering of 2.3° is achieved, satisfying applications such as collision avoidance for autonomous driving.

## Conflicts of interest

There are no conflicts to declare.

## Supplementary Material
